# Living with ongoing whiplash associated disorders: a qualitative study of individual perceptions and experiences

**DOI:** 10.1186/s12891-017-1882-9

**Published:** 2017-12-15

**Authors:** Carrie Ritchie, Carolyn Ehrlich, Michele Sterling

**Affiliations:** 1 Recover Injury Research Centre, NHMRC Centre of Research Excellence in Road Traffic Injury Recovery, University of Queensland, Herston, QLD 4006 Australia; 2 Hopkins Centre, Menzies Health Institute Queensland, Griffith University, Meadowbrook, QLD 4131 Australia; 3 Menzies Health Institute Queensland, Griffith University, Southport, QLD 4222 Australia

**Keywords:** Whiplash, Neck pain, Chronic pain, Qualitative, Thematic analysis

## Abstract

**Background:**

Whiplash associated disorders (WAD) are the most common non-hospitalised injury resulting from a motor vehicle crash. Approximately 50% of individuals with WAD experience on-going pain and disability. Results from intervention trials for individuals with chronic WAD are equivocal and optimal treatment continues to be a challenge. It may be that traditional quantitative measures included in treatment trials have not captured the full benefits patients experience through participation in an intervention. The aim of the present study was to explore participant subjective experiences and perceptions of living with on-going WAD.

**Methods:**

Twenty-seven individuals with chronic WAD participated in a one-on-one, semi-structured individual telephone interview. All interviews were audio-taped, transcribed verbatim and data were analysed using an inductive thematic analysis process.

**Results:**

Two themes emerged that described the experience of living with chronic WAD. First, all participants described navigating the healthcare system after their whiplash injury to help understand their injury and interpret therapeutic recommendations. Participants highlighted the need to ‘find the right healthcare practitioner (HCP)’ to help with this process. Many participants also described additional complexities in navigating and understanding healthcare incurred by interactions with compensation and funding systems. Second, participants described a journey of realisation, and the trial and error used to establish self-management strategies to both prevent and relieve pain. Participants described trying to understand the impact of their initial injury in relation to the gradual realisation that there may be on-going residual deficit. Seeking information from multiple sources, including personal experience gained through trial and error, was important in the search for acceptable management strategies.

**Conclusion:**

Recovery from a whiplash injury is an adaptive process and more than elimination of pain or disability, therefore may be different from common clinical patient reported outcomes. Early identification of patient understandings of pain, expectations of recovery, symptoms and therapy may help merge patient and HCP understandings. Additionally, helping individuals to recognise symptom triggers and develop appropriate strategies to minimise triggers may actively engage patients in their recovery. Finally, acknowledgement and validation of the whiplash injury by HCPs is seen by many as a necessary step in the recovery process.

**Electronic supplementary material:**

The online version of this article (10.1186/s12891-017-1882-9) contains supplementary material, which is available to authorized users.

## Background

Whiplash associated disorders (WAD) is the term used to describe a cluster of symptoms, including neck pain and disability, that typically result from an acceleration/deceleration movement of the neck following a motor vehicle collision (MVC). WAD are the most common non-hospitalised injuries resulting from a MVC, accounting for approximately 75% of all survivable MVC injuries [1]. Over the past few decades, recovery rates have remained unchanged with approximately 50% of individuals experiencing on-going pain and disability [2, 3]. Results from intervention trials for individuals with chronic WAD are equivocal and optimal treatment for these individuals continues to be a challenge [4–6]. It may be that traditional quantitative measures included in treatment trials have not captured the full benefits patients experience through participation in an intervention [7–9]. Qualitative methods provide opportunities to explore individual perspectives and gain insights into experiences and behaviours that may be intangible and difficult to measure quantitatively [10, 11].

Emerging qualitative data are providing valuable information about pain beliefs [12], coping [13], and recovery [14] for individuals with WAD. These data show that individuals with WAD have varied beliefs about pain and recovery, and some of these beliefs may be unhelpful to recovery [12, 14]. For example, a desire for restitution, or complete elimination of pain, reported by patients with WAD may be helpful in the acute injury stage but may be detrimental to recovery if pain persists [12]. While health care practitioners (HCPs) may be able to influence these beliefs [14], in one study, general practitioners (GP) appeared to conceptualize the management of WAD differently to their patients [15]. Given that individuals with WAD claim for allied health services as frequently as general practitioner visits, it would be beneficial to explore the alignment and interactions with a broader range of HCPs [16]. Gaining a more in-depth understanding of patient subjective experiences and the perceived effects of treatment may provide a deeper understanding of how pain and recovery fit into the lived experience. Additionally, integrating patients’ knowledge and experiences with health care is recognised as essential for the development of valid and relevant patient-reported outcome measures (PROM) [17] and clinical practice guidelines [18, 19]. Detailed information gathered about the lived experience may help guide the development of future suggestions for guidelines and treatments [7, 8]. The aim of the present study was to explore participant perceptions and experiences of living with on-going WAD through analysis of one-on-one interviews.

## Methods

### Participants

Participants were individuals, based in Brisbane, Queensland, Australia, who had previously completed a randomised controlled trial (RCT) titled ‘Comprehensive physiotherapy exercise programme or advice for chronic whiplash (PROMISE): a pragmatic randomised controlled trial’ (Australian New Zealand Clinical Trials Registry, number ACTRN12609000825257) [4] and who had agreed to be contacted for future studies (*n* = 70). From previous research using qualitative interviews in chronic pain [13, 14, 20], it was anticipated that a sample size of 18–22 would be required for the present study. A random selection of individuals from both the control and intervention groups of the RCT were invited to participate in this study. All participants provided either written or online (via a secure REDCap link [21]) informed consent prior to participating in the interview. Ethical clearance for the present study, including this consent process, was granted by Griffith University Human Research Ethics Committee (AHS/70/14/HREC).

### Previous RCT

Participants for the RCT were individuals who had experienced neck pain from a whiplash injury as the result of a MVC. Criteria for inclusion were: males and females between the ages of 18 & 65 years; WAD grade I (neck pain, stiffness or tenderness with no physical signs) or II (neck complaint and musculoskeletal signs that may include decreased range of movement or point tenderness) [22] of at least 3 months’ but less than 5 years duration; feeling at least moderate pain or moderate activity limitation because of pain; not receiving care for WAD (excluding medications); and proficient in written and spoken English. Individuals were excluded with known or suspected serious spinal disease (eg, metastatic disease of the spine), nerve root compromise (WAD grade III), confirmed fracture or dislocation at time of injury (WAD grade IV), or spinal surgery in the past 12 months.

The protocol for, and results of, the RCT have been published [4, 23]. Briefly, participants were randomised to receive either a comprehensive exercise programme (20 individually tailored and supervised exercise sessions) or advice (one exercise session and telephone support). All sessions were delivered by experienced physiotherapists who received training in the trial protocol. The results showed that the comprehensive exercise programme was no more effective than advice alone for the primary outcome of neck pain intensity (measured on a 0–10 numeric rating scale (NRS)).

### Data collection

One research assistant (CR), blind to participants’ outcome data, conducted semi-structured individual telephone interviews (each 30–40 min) using an interview guide designed to be flexible to ensure participants were able to fully express themselves [10, 11]. If needed, probes (in parentheses below) were used to ensure discussion of key topics. All participants were informed that it was their choice to respond or not to specific questions.

#### Interview guide

1. How well do you feel now? (WAD-related neck pain/disability, NRS)
2. Please tell me about any changes in your pain/disability and how you manage this since participating in the study? (management, activities- able and unable to do)
3. Why did you decide to participate in the research study? (previous treatment)
4. Do you have any thoughts on what might help individuals with neck pain/disability?
5. What does it mean to live well with a whiplash injury?

### Data analysis

All interviews were audio-taped and transcribed verbatim. Transcripts were uploaded to NVivo (Version 11, QSR International Pty Ltd., Doncaster, Australia), and data were analysed using an inductive thematic analysis process [24, 25]. This data-driven, analytic strategy was selected because it facilitated open exploration of individual experiences [24, 25]. Given that this type of data has not been previously reported, it was important to minimise the constraint of interpreting responses in relation to a pre-existing model or theory [24, 25].

Two authors (CR, CE) were involved in the initial data analysis for this study. CE is a Registered Nurse (current) with extensive experience in qualitative research methods, and CR is an exercise physiologist with some experience in qualitative research methods. Neither CE nor CR had experience clinically treating individuals with WAD, hence, bias regarding interpretation of treatment experiences was minimised.

Four steps were used in the analysis process. 1. Transcripts were checked for accuracy. 2 Two research assistants (RA) (authors CR, CE) openly read each transcript independently, summarised the content of the interview, extracted meaningful data on a response by response basis, and applied codes to categorise data. 3. The RAs met to check these codes and collapse these codes into broad themes by asking the question “what is this code an example of?” until all codes were parsimoniously accounted for. 4. It was acknowledged that these themes would be influenced by the RA’s experiences [24], hence, to maximise reliability and credibility of the results, an iterative process was used in which the RAs met regularly to discuss, review, revise and refine themes. The third author (MS), a physiotherapist with significant clinical experience in treating individuals with WAD, was involved in these regular discussions, reviews and revisions.

## Results

### Participants

Twenty-seven individuals completed interviews for this study (Table 1). While the interviewer remained blinded to participant outcomes measures from the original RCT during the interview, it became evident that 13 participants had been in the treatment group and 14, the control group. Following the qualitative analysis for the present study, data were re-visited from the original RCT. Twenty participants had been involved in a compensation claim: seven had a worker’s compensation claim, 12 had Compulsory Third Party (CTP) claims and one had both. CTP insurance in Queensland is a common law, “fault” based scheme. Compensation for injury caused by a motor vehicle is covered by CTP only if the injured person can establish that the other motorist is at fault.

**Table 1:** Participant characteristics

	Participants *n* = 27
Mean Age (SD)	53 years (13)
Female gender (%)	63% (*n* = 17)
Mean Duration post whiplash injury (SD)	77 (15) months
Mean NRS- present study (SD)	3.8 (2.1)
Number of participants with minimal or no pain (NRS ≤ 2)-present study	9
Previous RCT data	
Mean NRS- 12 months post RCT (SD)	3.9 (2.2)
Number of participants classified as responders at 12 months post RCT (2 unit improvement on NRS from baseline)	12

SD: standard deviation

NRS: numeric rating scale: 0(no pain) to 10(worse pain ever)

Of the additional 43 Brisbane-based participants who agreed to be contacted for future studies, five did not wish to participate and 38 were not contactable (telephone not in service = 12; left message twice with no return response = 18; did not answer and no facility to leave a message = 8).

### Living with on-going WAD

Participants described two main processes that impacted their experience of living with chronic WAD. First participants described the need to navigate, interpret and understand individual care and treatment in the context of healthcare systems. For some participants, there was also an identified need to somehow make sense of healthcare within compensation and funding systems. Second, participants discussed the ‘journey’ from an acute injury experience to one with residual deficit and ongoing symptoms which needed to be assessed, understood and managed to both prevent pain and to relieve symptoms.

Quotes provided within the text include participant number, sex, and either C (control) or I (intervention). Additional quotes are provided in the supplementary material (Additional file 1: Table S1).

### Theme 1: The healthcare and compensation systems – A new experience to be navigated, interpreted and understood

All participants described navigating the healthcare system after their whiplash injury to help understand their injury and interpret therapeutic recommendations. Two sub-themes arose from these discussions. First, participants highlighted the need to ‘find the right healthcare practitioner (HCP)’. Second, many participants described additional complexities in navigating and understanding healthcare incurred by interactions with compensation and funding systems.

#### Subtheme 1a: Finding the ‘right’ HCP and the impact of validation and matched expectations on navigating the healthcare system

A ‘good’ HCP was considered essential by most participants and was characterised as someone who believed the participant and validated their experience, explained things well, and provided a constructive management approach that matched the individual’s beliefs and expectations. With the support of a ‘good’ HCP, participants gained confidence in navigating the healthcare system and understanding their whiplash injury. Without the support of a HCP, participants felt confused about their injury.

*“My doctor said to me it’s about time I faced up to the fact that there’s nothing wrong with me, there’s nothing physically wrong. He suggested that I go to a psychiatrist or a psychologist for treatment of the mental side of things and that once that was treated then I would be right. The sooner I’ve faced up to that then the better off I’d be.” (P17, M, I)*

A mismatch in beliefs and expectations about recommended management strategies led individuals to interpret recommendations as incorrect and inadequate.

*“When you go to a GP [general practitioner] for that sort of an issue, he goes, well, you need an MRI [magnetic resonance imaging], and it doesn’t. I don’t really need any more MRI’s.” (P22, M, C)*

As a result, participants explained a process of ‘shopping around’ to find a ‘good’ HCP; a process that was characterised by a sense that it was lucky to find a ‘good’ practitioner.

*“I saw a number of different practitioners and I guess I found the person now that I see who has had the best result with me. So I guess it was just for me finding the right match. I went to a lot of different physios [physiotherapist], muscular-skeletal people and didn’t have good results.” (P23, F, C)*

A couple of participants explained that it was not always possible to gauge initially whether or not the healthcare received was ‘good’ until a HCP was found who the participant believed provided a better care experience. Not finding a ‘good’ HCP initially was believed to contribute to prolonged non-recovery.

*“The physio [physiotherapist] that I first saw was fairly young and even though she did have quite an experienced supervisor who said that she understood whiplash injuries, I really don’t believe that as a practice, they understood whiplash injuries. If I had seen my current physio from day one, there is potential that I would not have spent years trying to get back on top of things.” (P6, F, I)*

Interestingly, several participants expressed relief at finally finding a ‘good’ HCP through the RCT.

*“He [RCT physiotherapist] was absolutely wonderful and knowing that people were seriously looking at what was going on took the stress out of the situation for me and just gave me other options of how to move and sit and that sort of thing.” (P21, F, C)*

#### Subtheme 1b: Complexities incurred by interactions with compensation and funding systems

Several participants felt that compensation systems were focussed on monetary cost at the expense of optimal, individualised care. Although it was not the purpose of this study to discuss the details of compensation systems, participants discussed a belief that the perceived cost motivation of compensation systems may have driven recommendations for early return to work and insufficient treatments.

*“Immediately after the accident, it was a [worker’s compensation] thing, and I did go to the physio. But I think [worker’s compensation] was inclined to make you get better within a short space of time. I was being pushed very much by [worker’s compensation], they wanted all their costs signed off within a few months. So they were very impatient about the idea that I might have wanted to do something ongoing.” (P9, F, C)*

In addition, participants discussed the difficulties incurred when required to consult HCPs specified as Independent Medical Examiners (IME) by the compensation system. The varied opinions and different treatment recommendations exacerbated difficulties in navigating the healthcare system. Two participants were still going through legal processes associated with unsettled compensation claims.

*“I had tried to succeed in getting an insurance claim and in doing that they want you to see your own doctor and then to see their doctor. You know, it was hard, and the insurance company and their doctor, really the pair of them weren’t much help. They reckoned if I could walk around I was all right. Well I could walk around because I usually walk around many times faster than that.” (P18, M, I)*

Cost of care was mentioned by a number of participants. Some treatments that participants believed were beneficial were sometimes unaffordable and participants were required to navigate the healthcare system within the constraints of monetary cost. While one participant only used treatments available through her private health care, for others, the inability to access care believed to be beneficial was thought to affect recovery.

*“I had physiotherapy for I think six months but I couldn’t afford each session. The free ones were fine but I couldn’t afford the $50 hit each time to go further, see what I mean, so I then started to use a massage because I could afford that every now and then.” (P25, F, I)*

### Theme 2: Understanding the initial injury and moving from acute injury to chronicity – A journey of realisation and trial and error to establish self-management strategies to both prevent and relieve pain, symptoms and disability

Many participants described living with residual chronic pain which was a constant reminder of their whiplash injury. Participants were not able to do everything that they were able to do prior to injury. This ongoing deficit was physically and mentally wearing, and meant that managing the psychological impact of chronic pain was important. Two sub-themes arose from these discussions. First, participants described trying to understand the impact of their initial injury in relation to the gradual realisation that there may be on-going residual deficit. Second, participants described the importance of seeking information from multiple sources, including personal experience gained through trial and error, in search of acceptable strategies to both prevent pain and to relieve symptoms.

#### Sub-theme 2a: The impact of the initial injury and the gradual realisation of chronicity

Participants described the time taken to understand the impact of their initial injury. It was not until some time post-injury that participants realised they were unlikely to regain their pre-injury state. Participants described how they learned to live with ongoing deficit rather than trying to achieve pain-free recovery.

*“It’s [pain]still there but at four [rating NRS]you can manage it. You can forget about it even. You can get on and do things because you’ve become so used to it, and if you take a bit of Panadol[paracetamol] that kind of helps. It [medication] just takes the edge off it.” (P19, F, I)*

Some participants felt that HCPs did not provide accurate information early post-injury.

*“Well the doctor said it’s [neck pain] going to go away in six weeks, so I just thought “I’ll wait six weeks and it’ll be gone,” but if someone would have said, “Well it’s [neck pain] actually more a long-term thing and you could have it for the rest of your life possibly,” then I would have taken it a bit more seriously.” * (P15, F, C)

Two participants acknowledged their own complacency based on the perceived mild severity of the MVC. Several participants stressed that this lack of initial awareness resulted in insufficient early management and consequently impacted their longer-term recovery.

*“At the time I didn’t think much of it [neck pain], there wasn’t a lot of damage to the car or anything and I didn’t follow up on it. Had I known that I would still have neck pain down the track, I would have taken it more seriously.” (P4, M, I)*

#### Sub-theme 2b: Sourcing information and trial and error to find effective self-management strategies to both prevent and relieve pain

Participants acknowledged the need to learn to identify: symptoms, activities that exacerbated symptoms, and the impact of various strategies on these symptoms. Participants continually scanned for pain and took care to avoid pain. Information was cobbled together from multiple sources in search of useful treatments and strategies. Several participants indicated that they could not always rely on advice from their doctor and explained that some doctors only wanted to prescribe medication or provide referral for unnecessary tests. In addition, some doctors thought physiotherapy interventions were appropriate treatments for whiplash, while other doctors disagreed. As a result, participants did not always follow their doctor’s advice and often felt they had to rely on their own beliefs and experience.

*“It was trial and error and I guess the realisation that there was nothing available to actually help, so I just got on about trying to work out how I was going to manage and live with it.” (P22, M, C)*

Participants described a continual search for something that worked and many participants were willing to try anything.

*“I was given painkillers and Voltaren creams [diclofenac] and exercises and I’ve tried everything since. I’ve done dry needling, I’ve done osteo [therapy], I’ve done everything.” (P11, F, C)*

Based on these experiences and within the context of personal beliefs, specific strategies were adopted to prevent pain and relieve symptoms. Participants highlighted the motivation to implement regularly specific strategies to keep pain away.

*“You learn to live with it. You know the pain is there, you are aware of it. I do the exercises, I do whatever I can to prevent it because I don’t like pain and so I know when to stop doing things so not to continue the pain.” (P5, F, C)*

The most discussed adaptive strategy was limiting time spent on the computer or driving. For several participants this meant using a timing device as a reminder. Many participants also detailed the need for specific pillows and mattresses to facilitate sleep, a measure of the impact of their whiplash injury. Neck-specific exercises were used by different participants to either prevent or relieve pain. Exercises included adaptations of those learned through involvement in the RCT or from other HCPs.

*“I have a series of exercises, physio [physiotherapist] type exercises that I can do to manage the condition. I do them most days, but I have to admit it’s one of those things that when there’s no pain, there’s nothing to remind me to do them.” (P13, M, I)*

For relief of pain, heat packs and neck-specific exercises were two strategies used by many participants. Additional strategies included massage and assistance from various HCPs such as physiotherapists and chiropractors. Two participants used HCPs as a preventive strategy: one had quarterly massages to prevent pain and one visited a physiotherapist twice a year to ensure correct posture and loosen muscles to avoid neck pain. Early symptom detection was important.

*“I get headaches from the problems with my neck but I know what to look for and then I go straight for a massage and if it gets too bad I go back to physio [physiotherapist], I’ve learned to manage it.” (P24, F, C)*

Part of this process was to make choices by weighing up the pros and cons of actions that might exacerbate pain.

*“Certain activities I don’t do any more at the gym, and pushbike-riding also is a bit of a challenge from time to time. It is what it is, so I either make a conscious decision to participate and live with the consequences of it, or just do it to a smaller level than I would’ve done in the past.” (P22, M, C)*

## Discussion

Participants in this study had been living with WAD for an average of 6.5 years and continued to find it challenging and exhausting. Living with residual deficit was described as more than trying to eliminate pain and disability. Although the journey from acute to chronic WAD was unique to each individual, two key themes described this journey. First, there was a need to navigate healthcare systems in search of a HCP who validated their injury and provided information and therapeutic strategies that matched each individual’s personal beliefs. Oftentimes this process was complicated by requirements of compensation systems, and, for some, constrained by monetary costs. Second, individuals described the journey of gradual realisation that that their injury may be on-going, and the consequent process of trial and error to find the best sustainable strategies to both prevent and relieve symptoms. These qualitative data provide: insight into living with chronic WAD; and resultant suggestions for treatment guidelines and for HCPs working with individuals with WAD.

The importance of being believed and validated aligns with previous literature about patient experiences with chronic pain [20, 26, 27]. There is no diagnostic test for WAD so patients may feel a need to prove the existence of their pain resulting in feelings of being judged about the legitimacy of their injury [20, 27, 28]. It has been proposed that patients with chronic low back pain become suspended in a chronic pain sick role until legitimacy is established [28]. One suggestion to help legitimise pain is to include a biomedical explanation of pain [28]. Accumulating evidence indicates a biopsychosocial model of recovery for individuals with WAD [3, 29, 30], and first line treatment guidelines include reassurance, and encouragement to stay active and return to usual activities [31]. Patients who are experiencing new and acute pain with usual activities may be confused by this recommendation to return to these activities, and subsequently may feel misunderstood. Reassurance and an optimistic outlook are important, however helping patients to understand pain processes and recovery trajectories may add to these recommendations and increase the likelihood that patients feel understood and believed [12, 14, 19]. Several participants in the present study, including three participants who had been in the single session control group, highlighted a sense of relief when the physiotherapists involved in the RCT clarified pain processes and recovery from WAD. The HCPs involved in the RCT underwent specific training and were provided with biomedical and psychosocial resources. An explanation of pain processes was provided to participants as a small part of both the single control group session and the intervention. Providing resources to HCPs regarding specific pain processes, increasing awareness that biomedical explanations about pain processes may be valued by patients, and providing strategies within clinical guidelines on how to include these within a more contemporary biopsychosocial model of care may help patients to feel believed and validated.

Strategies to legitimise pain may be particularly important for individuals with WAD since many are involved in compensation systems and risk being considered a malingerer [12]. Twenty participants in the present study were involved in compensation processes. Although it was not the specific purpose of the present study to ask about experiences with compensation systems, many participants voluntarily discussed complications in navigating healthcare when simultaneously involved with compensation systems. Previous qualitative studies indicated that HCPs believed that patients involved in compensation claims were exposed to a conflict of interest between desired recovery and the perceived need to show disability to receive compensation [32], and general practitioners treating individuals with WAD were reported to be reluctant to become involved with compensation issues [15]. Possible mixed messages from HCPS in addition to the fact that patients are sometimes required to consult with unknown IMEs may increase confusion in navigating and understanding healthcare. Further exploration of factors associated with the intersection between healthcare within and outside of the compensation systems may help identify factors addressable in the clinical environment.

Individuals who feel believed and understood are more likely to actively engage with practitioners about management decisions [20, 26]. Furthermore, positive interactions (eg therapeutic alliance) have been associated with better perceived treatment effects, and reductions in pain and disability in LBP patients [33]. It appears that at some stage, participants in the present study effectively engaged with HCPs. Several participants in the present study regularly performed specific neck exercises learned from HCPs, and several participants continued to seek treatments such as massage and physiotherapy from HCPs. Although effect sizes are small, systematic reviews have concluded that exercise programs and multimodal physiotherapy are the most effective non-invasive treatments for patients with chronic WAD [34, 35]. It is promising that the treatments received by participants in the present study mostly match with evidence to date.

A process of trial and error by participants was used to identify suitable strategies. Specific but different strategies were employed to prevent pain or to alleviate pain. Establishing these strategies took time, and several participants felt that an early lack of awareness of the potential for on-going pain led them to underestimate the importance of early management and ultimately contributed to their chronic condition. Individuals with acute WAD have also expressed a desire for more realistic expectations of recovery [14]. Current guidelines recommend assessing expectations of recovery with the question “Do you think you are going to get better soon?” [31]. For patients with a poor expectation of recovery, the recommendations are for ‘further psychological assessment and consideration of referral to a clinician with expertise in the management of WAD’ [31]. The results of the current study suggest that it is also important to identify patient understanding of recovery, and whether or not expectations of recovery are realistic. Given the heterogeneity of WAD recovery, a patient-centred care approach appears to be important. Patient-centred care refers to an equal partnership between the HCP and patient and recognises that a good outcome equates to what is important to the individual patient [36, 37]. Adapting guidelines to emphasise early consideration of patient’s understandings of pain and recovery, in addition to expectations of recovery may help provide a foundation for a mutually acceptable approach to therapy.

While the qualitative data explored in this study add to traditional quantitative measures to capture a more complete understanding of patients’ experiences of living with on-going WAD, there are several limitations to this research. First, participants for this study were from Brisbane, Australia. It is acknowledged that patient experiences with a whiplash injury may vary in different countries and jurisdictions. Second, participants for this study had volunteered for the previous interventional RCT. This cohort may have found recovery particularly difficult and therefore sought out alternative strategies, or may have been more proactive in searching for treatment. Although, some caution is needed in generalising these data, the resultant heterogeneity of the sample population in this study (although not a deliberate design intention) potentially improves the transferability of our findings and is a strength of this study. Finally, the results were not re-presented to participants to check that the researcher understanding was consistent with participant experience. However, reliability and credibility of the results were maximised through the iterative process used to review, revise and refine themes and the broad range of researcher backgrounds.

## Conclusion

In conclusion, thematic analysis indicated that recovery from a whiplash injury is an adaptive process and more than elimination of pain or disability, and therefore may be different from common PROMs. Acknowledgement and validation of the whiplash injury by HCPs is important and requires more than simply providing current guideline recommendations of reassurance, and encouragement to stay active and return to usual activities. A patient-centred care approach is needed to try and identify patient understanding of pain and recovery, and expectations of recovery, symptoms and therapy. Additionally, helping individuals recognise symptom triggers and develop appropriate strategies to minimise triggers may actively engage patients in their recovery. A merge in patient and HCP understandings may help provide a foundation for a mutually acceptable approach to therapy.

## Additional file

Additional file 1: Table S1. Additional quotes to support the themes and sub-themes. Table with additional quotes to support the sub-themes and themes. (DOCX 26 kb)

## Abbreviations

C: Control group participant
CTP: Compulsory Third Party
F: Female
GP: General Practitioner
HCP: Healthcare Practitioner
I: Intervention group participant
IME: Independent Medical Examiner
M: Male
MRI: Magnetic Resonance Imaging
MVC: Motor Vehicle Collision
NRS: Numeric Rating Scale
PROM: Patient Reported Outcome Measure
RA: Research Assistant
RCT: Randomised Controlled Trial
WAD: Whiplash Associated Disorders
