# Supplementary material for: Living with ongoing whiplash associated disorders: a qualitative study of individual perceptions and experiences
Source: BMC Musculoskelet Disord. 2017 Dec 15;18:531. doi: 10.1186/s12891-017-1882-9 (PMC5732403; doi:10.1186/s12891-017-1882-9)
Supplement: Additional file 1: Table S1. — Additional quotes to support the themes and sub-themes. Table with additional quotes to support the sub-themes and themes. (DOCX 26 kb) [file 12891_2017_1882_MOESM1_ESM.docx]

Additional file 1: Table S1. Additional quotes to support the themes and sub-themes.

|  |  | Participant number, sex, and C (control group) or I (intervention group) |
| --- | --- | --- |
| **THEME 1**  **The healthcare and compensation systems – a new experience to be navigated, interpreted and understood** | | |
| Subtheme 1a: Finding the right healthcare practitioner | *Don’t listen to the doctors, because the doctors aren’t big in believing in physios, they tend not to work together which is a bit of shame but I think it’s really important to find the right physio [physiotherapist], she was good for me physically and mentally. You just need to get the right person that knows what to work on for you.* | P24, F, C |
|  | *Don't feel sorry for yourself, just get out there and know that there are people who will listen to you, who say “Yes, yes it’s a bad thing that happened but exercise will help you”.* | P8, F, C |
|  | *It all depends on how proactive your doctor is, it all depends on whether you find the right physio [physiotherapist], it all depends on whether you accept that you need psychological intervention, there’s so many variables there.* | P6, F, I |
|  | *I've just felt a huge improvement. I did have a very good physio [physiotherapist]. I was very lucky.* | P12, F, I |
|  | *I just happened to come across a physio [physiotherapist] who does acupuncture and he’s inspirational. We sort of gelled on that.* *It was just sheer luck. Sheer luck or karma, take your pick.* | P8, F, C |
|  | *I feel like you just get moved from one specialist to another and they all just do their little thing and move you on and charge you a lot of money, whereas [my chiropractor] that I see very much does everything or then refers me if he thinks I need to, so it’s more complete, which I found a lot better for me.* | P23, F, C |
|  | *It’s a slow progress and you’ve just got to keep knocking them off one by one and it would be nice if the medical profession was onside. There were different physios [physiotherapists], some physios knew what they were doing and other physios didn’t know what they were doing. Some people didn’t have a clue.* | P17, M, I |
|  | *I'd been to the doctors and they said everything was all right, but it really makes me angry and here I am I can't even get a decent night’s sleep.* | P18, M, I |
|  | *I don’t know if there’s different physiotherapists or what, I had one physiotherapist and for some reason she just didn’t work, I don’t know what happened but I ended up going to a second one and she was really good, she was better for me.* | P25, F, I |
|  | *He [RCT physiotherapist] made me feel less as if I was imagining things. I knew that it is something that you could have an ongoing problem with, and I wasn’t just being silly.* | P9, F, C |
|  | *Most of them [physiotherapists] would just treat you, but not actually give you exercises to do, where he [RCT physiotherapist] did a programme that was exercise related. It was a big part of it, so that was sort of different.* *He was very, very good.* | P12, F, I |
| Subtheme 1b: Complexities incurred by interactions with compensation and funding systems | *Insurance companies are very good at sending you off, or at least that’s what happened to me, because [insurance company] paid, they sent me to a neurologist, they sent me for an MRI, they sent me for every scan you could possibly imagine.* | P6, F, I |
|  | *Employers will lie to the employees and say, “You are required to go to our doctor.” And when I found that the company that I worked for would regularly send employees to a doctor that they knew was going to to falsify downways. Companies are sending people back to work far too soon and people are getting exacerbation injuries. It was made worse by going back too early.* | P26, M, I |
|  | *Because it was through [worker’s compensation] at one point and that came to nothing only because I went back to work and they saw that as being fine, when it wasn’t, it’s just that I couldn’t afford not to. So I went back to work out of desperation and so they saw that as being able to work when it’s not.* | P11, F, C |
|  | *I was actually criticised by the insurance company as trying to shop around until I found a diagnosis that agreed with what I believed. What are you supposed to do when you have health professionals telling you that there’s nothing wrong with you it’s all in your mind?* | P17, M, I |
|  | *It’s a minor injury is the bizarre part especially when you’re trying to prove to the insurance company that you need intervention because on paper there was nothing wrong with me* | P6, F, I |
|  | *I can handle a lot of pain but I think I’m being penalised by my lawyers because they don’t particularly believe what I am going through, they said oh no I’ve got mental illness. Well I feel that that phrase has a blight on my name.* | P3, M, C |
|  | *I had been to physio before over a period of a few weeks. It was something like seven weeks and that was all the insurance would cover me for and it wasn’t bad while I was going but as soon as I stopped I had lots of problems.* | P24, F, C |
|  | *All muscular-skeletal is extremely expensive and I guess I’m a young single mum so I’ve got to be careful with-- like it was $200 a visit and something like that. It’s really hard when you are dealing with your health and stuff, and so that’s where I started to get a bit “Oh, it’s so much money and I feel like I’m going from one person to the next” and that’s when I kind of stopped it all a bit and just went okay, let’s just try something different and go a bit more natural.* | P23, F, C |
|  | *So my choices with my budget were well my kids come first and so then I probably wasn't getting the treatment I should’ve been.* | P8, F, C |
|  | *I don't have a lot of time to run out and money on the side to just go and get relief with acupuncture around the clock so if I find I do get the tightness and the headaches and I find that same knot and lump there, I try to press it round. Jeez, if I had more money I’d have it [acupuncture] done regularly but it all comes down to money at this stage.* | P7, F, I |
|  | *It would have been good to go [to hydrotherapy] twice a week at least, I think. But the twice a week would cost me $27.00 a week.* *And when you’re on a pension, $27.00 a week every week that’s working out at $1300 a year.* | P16, F, C |
| **THEME 2**  **Understanding the initial injury and moving from acute injury to chronicity – a journey of realisation and trial and error to establish self-management strategies to both prevent and relieve pain, symptoms and disability.** | | |
| Subtheme 2a: The impact of the initial injury and the gradual realisation of chronicity | *I think it’s maybe just to get that understanding that it won’t go away very quickly. For me, I’m very fit and healthy and that was probably the hardest thing to mentally deal with, that this could be something that’s quite long-term and I’d never had a long-term injury.* | P15, F, C |
|  | *Does it hold me back from exercise? Well, I don’t swim like I used to. Does it stop me doing all forms of exercise? No, it doesn’t so I guess I’ve adapted.* | P4, M, I |
|  | *You’re just going to have to live with it and find your own way to live with it. If I had a button that could switch it off, I would, but I can’t, so what can I do, you know? I’ve just got to work and do the best I can.* | P11, F, C |
|  | *It was very hard getting information that I needed. I’m not criticising anybody here, I’m just saying that if there was a reputable, credible source that you could go to and say I have whiplash, I’m dizzy all day, I can’t get out of bed. Someone could say, try this program, this will work. Then I would have saved myself probably a whole year maybe in my recovery.* | P6, F, I |
|  | *I think learning to manage it and learning to accept that it’s there is hard. Accepting that it’s there is the hardest thing and accepting that I can’t function as well as I ought to be able to.* | P10, F, C |
|  | *I probably didn't expect it to be, it’s not only physically wearing, I find it mentally wearing, you just wish it would go away. I can say it’s an absolute pain in the backside to be honest. It’s very, very wearing. I can’t think of any other way to describe it. I would like to wake up, bounce out of bed and things like that but I don't.* | P21. F, C |
|  | *I mean for me see it was a long process. I mean it was a couple of years for me. I mean my neck was shocking at the end of the day my neck will never be exact to what it was, that’s just reality for me, but I can manage it now and that’s the difference.* | P24, F, C |
|  | *If I had seen [physiotherapist] from day one, there is potential that I would not have spent years trying to get back on top of things. Anyway, easy to say with hindsight* | P6, F, I |
|  | *If I had been afforded what I wanted, a couple of hours a day [of physiotherapy] then maybe it would have given the muscular or nervous system enough time to relax and then be able to heal.* | P3, M, C |
|  | *I didn't get it treated probably at the right time I’d say, so I’m stuck with it. I wish that the doctors would probably if anything would put people through treatments or give some form of something. I can’t say enough that if I’d had intervention early. That’s the hardest thing, it really got me cranky for a long time* | P7, F, I |
|  | *The medical profession did very little to investigate if there were any other issues [shoulder pain]. It’s only through persistence that the other issues are slowly getting fixed. It would’ve been nice to have been fixed at the time.* | P17, M, I |
| Subtheme 2b: Sourcing information, trial and error and self-management | *I’ve still got to be careful with my neck. I still get problems and I get shooting pains and I still have problems with my neck and I get headaches but I know what to look for and then I go straight for massage and if it gets too bad I go back to physio. So, yeah, I’ve learnt to manage it.* | P23, F, C |
|  | *You just need to know what makes it flare up and just be aware of that and not get upset when it does but know how to manage it a bit better, like self-manage.* | P15, F, C |
|  | *I have learned to identify the symptoms I guess a bit better and I guess, try new things. I never thought that I would be able to lift weights or anything but it’s just a case of going to try and it and that wasn’t so bad so you keep doing it.* | P4, M, I |
|  | *A lot of it is a self-awareness thing and working out what works for you whether that be physically or mentally and strategies. After the accident I had the chiropractor treatment, I went to physios, all sorts of things.* | P21, F, C |
|  | *I ended up taking a lot of time off work and I went and had lots of more physio [physiotherapy] treatment and I was going to a number of different people, one for acupuncture, I was getting massage, and then I started doing Pilates with one of them, and I was just doing lots of things to try and work out the best thing to help me.* | P19, F, I |
|  | *I was prepared to try anything and I even tried other alternative therapies as well, anything I could do to get my life back.* | P24, F, C |
|  | *After a certain length of time you end up with a really tight neck and you’re a mess and you try to sleep that night and it’s not worth it, so you need something that relaxes your muscles. It’s trial and error I suppose.* | P7, F, I |
|  | *There have been times where I’ve then gone to a sports physio [physiotherapist] and really just tried to work on posture and those sorts of areas, but not as a result of any direction from a GP or someone like that, it’s more a result of OK, what, what else might I be able to try to resolve this?* | P22, M, C |
|  | *I was really exasperated and just desperate for anything, any kind of help and there wasn’t much out there so I just felt a bit lost with what to do or where to go and so I just thought the research [RCT] was just another avenue that might help.* | P23, F, C |
|  | *I had to learn the hard way about propping myself up with pillows. Using full length body pillows to, you know, keep and not--to lie perpendicular with your spine, so that one leg is on top of the other leg.* | P26, M, I |
|  | *You learn to live with it. You know the pain is there, you are aware of it. I do the exercises, I do whatever I can to prevent it because I don’t like pain and so I know when to stop doing things so not to continue the pain.* | P5, F, C |
|  | *I go to the gym -- I thought I could never do that again, like do more weight lifting and everything. I do pump classes because I lost a lot of muscle tone on that side, but I’ve been slowly able to rebuild that up but I am very conscious about what I do* | P23, F, C |
|  | *I do some stretches and it then tends to release itself, it tends to get better.* | P9, F, C |
|  | *I still do the exercises. At the end of the programme [RCT] we worked out a shorter routine, and I still do that every day.* | P12, F, I |
|  | *I do find that regular exercise, and when I say exercise I mean with weights, weights and theraband sort of exercise that physios and that have given me over the period of time, I do find that they help. If I keep those up regularly I find that I am, I cope quite well. But if I don’t, if I miss those for more than a few days I start to get a lot of pain in my shoulder and everything tightens up and everything like that.* | P18, M, I |
|  | *Posture’s very important in keeping the pain away. So I do exercises for my posture and try to just hold myself up whenever I think about it, up straight. Because that is, I mean if I start slouching I do get more pain.* | P19, F, I |
|  | *It’s [RCT] given me a series of exercise, physio type exercises that I can do to manage the condition particularly when it’s bad in cold weather.* | P13, M, I |
|  | *I was doing the exercise and stuff and the headaches were bad but now that it’s only very infrequently I’ll just go to the physio because my private health covers all that so I just go there and that sorts it for good.* | P20, F, C |
|  | *I have an email in my inbox here. It says neck exercises and I’ve got it set to snooze for an hour, so every hour it pops up and tells me to do my neck exercises and I spend two or three minutes doing my neck exercises here at my desk and I hit it to snooze again for another hour* | P13, M, I |
|  | *I’m looking after myself and how I set-up my work station, taking regular breaks.* | P1, F, C |
|  | *I’ve always self-managed my pain, I sort of know what works for me, and doing the job I’m doing, I’m doing phone work and I’m very conscious of how I sit, how I move and if I feel that all of a sudden I think everything’s locking up, I get up and do my stretching exercises and walk round and stuff like that.* | P21, F, C |
|  | *I was just like looking for a quick fix and wanted it to go away and it was frustrating but now, yeah, if it starts to hurt I kind of just move away from the computer.* | P15, F, C |
|  | *Sitting too long at the computer. That’s probably the one thing that does it [increases pain] or sitting too long in one position, either at the computer or at the sewing machine. Sometimes I linger longer than I should, but at least it- and I do actually set the alarm now to make myself get up.* | P10, F, C |
|  | *I still can’t drive long distances just because I can’t sit for long periods of time so any activity that involves one position for a long period I need to break up so I can now sit for just over 45 minutes.* | P6, F, I |
|  | *I’ve found in the last 12 months that I can’t sit in the car for any longer than two hours.* | P10, F, C |
|  | *I get a one or a two now and then, and I just simply increase my exercises again and concentrate on moving my neck around very gently. Not overdoing it. I'm thinking, oh maybe that pain’s there because I've been on the computer too much or staying still too much. So I move but I do it gently.* | P18, M, I |
|  | *I try and get up in the intervals. A heat pack - probably every week there’s a day where I might use a heat pack. I try to do and keep up exercises to keep it limber* | P11, F, C |
|  | *Heat packs help and massage, that really helped.* | P7, F, I |
|  | *I tend to go for massage and I still use the hot pack, hot packs I found quite good.* | P24, F, C |
|  | *The best thing I find for it is the heat. So I quite often sit with a hot water bottle by my neck, that sort of thing, and that is very comforting.* | P9, F, C |
|  | *I just don’t really do anything apart from maybe a heat pack occasionally but other than that, you just have to live through it because there’s not really much--I don’t really wish to spend more money on trying to fix it or anything.* | P15, F, C |
|  | *I’ll put either a cold pack or a heat pack at the back of my neck when I can feel it tensing up* | P21, F, C |
|  | *I do have good quality pillows. I have invested in that and I do take them with me when I go places. I’m aware that something as simple as the wrong pillow or sleeping funny will affect it, so I’m conscious of all these things all the time* | P23, F, C |
|  | *I do is I have a full length body pillow and I have other pillows. So what I do is I prop myself up in bed each and every night. I get myself into a position and try not to move too much.* | P26, M, I |
|  | *I have tried numerous types of pillows – because I need a low pillow if I’m lying on my back but if I turn over to lie on my side it needs to be higher. And that is difficult* | P19, F, I |
|  | *I find the massage, the acupuncture, that deep tissue works a lot more for, and also osteo sometimes is good. I guess I saw so many people.* | P23, F, C |
|  | *I see a remedial therapist at least once every 6 weeks and a massage therapist and I see a physio about once every 6 months and that’s to do with my neck. It’s a result of the whiplash injury but more as a prevention – like as soon as I notice things, I go straight away instead of waiting for it to get to the point where I can’t even move my head.* | P1, F, C |
|  | *I still rely on the physio. I know if I say to him “I have a problem” he will spend as much time with me as we need to.* | P8, F, C |
|  | *Some massage stirs it up more so you really have to have someone who really knows what they’re doing, and I’ve learnt this over the years.* | P19, F, I |
|  | *I go to a hydrotherapy exercises now which is probably helping with the rest of my back as well.* | P16, F, C |
|  | *One of the things that has been very handy has been standing there in the shower with hot water running on the back of my neck for a while to relax muscles and that started the day and just loosening up the neck muscles.* | P13, M, I |
|  | *I don’t have the same mobility in my neck as somebody else does. But I keep going and take analgesics for the pain and just keep going.* | P4, M, I |
